# Comprehensive immunohistochemical analysis of tumor microenvironment immune status in esophageal squamous cell carcinoma

**DOI:** 10.18632/oncotarget.10055

**Published:** 2016-06-15

**Authors:** Ken Hatogai, Shigehisa Kitano, Satoshi Fujii, Takashi Kojima, Hiroyuki Daiko, Shogo Nomura, Takayuki Yoshino, Atsushi Ohtsu, Yuichi Takiguchi, Toshihiko Doi, Atsushi Ochiai

**Affiliations:** ^1^ Division of Pathology, Exploratory Oncology Research and Clinical Trial Center, National Cancer Center, Kashiwa, Chiba, Japan; ^2^ Department of Gastroenterology and Gastrointestinal Oncology, National Cancer Center Hospital East, Kashiwa, Chiba, Japan; ^3^ Department of Medical Oncology, Graduate School of Medicine, Chiba University, Chuo-ku, Chiba, Japan; ^4^ Department of Experimental Therapeutics, Exploratory Oncology Research and Clinical Trial Center, National Cancer Center, Tokyo, Japan; ^5^ Department of Esophageal Surgery, National Cancer Center Hospital East, Kashiwa, Chiba, Japan; ^6^ Biostatistics Division, Center for Research Administration and Support, National Cancer Center, Kashiwa, Japan; ^7^ Exploratory Oncology Research and Clinical Trial Center, National Cancer Center, Kashiwa, Japan

**Keywords:** esophageal cancer, immunohistochemistry, PD-L1, tumor infiltrating lymphocyte, macrophage

## Abstract

Immunotherapy with anti-PD-1 antibody preliminarily showed promising efficacy for treating esophageal squamous cell carcinoma (ESCC). Herein, we used tissue microarrays and immunohistochemically analyzed PD-L1 and various tumor infiltrating immune cells (TIICs) in specimens from 196 ESCC patients who had undergone curative resection without preoperative therapy. PD-L1 expressions in tumor cells (TCs) and TIICs, as well as infiltration of lymphocytes (CD4^+^, CD8^+^, FOXP3^+^, and PD- 1^+^) and macrophages (CD68^+^ and CD204^+^), were evaluated. PD-L1 was expressed in TCs of 18.4% and in TIICs of 83.3% of these patients. PD-L1 expressions in TCs and TIICs were associated with significant infiltration of various TIIC types, especially CD8^+^ cells. PD-L1 expressions in both TCs and TIICs were significantly associated with favorable overall survival, and combining their levels enhanced prognostic accuracy. Prognostic impacts of PD-L1 expressions in TCs and TIICs, abundant PD-1^+^ cell infiltration, a high CD8^+^/FOXP3^+^ ratio, and the CD8^+^/CD204^+^ ratio remained significant after adjusting for clinicopathological factors. In conclusion, PD-L1 expression reflects anti-tumor immunity, and PD-1/PD-L1 expression and the ratio of infiltrating effector to immune suppressor cells have prognostic value. Therapeutic strategies inhibiting the PD-1/PD-L1 signal and immune suppressor cells are anticipated in ESCC patients.

## INTRODUCTION

Squamous cell carcinoma is the predominant histological type of esophageal cancer worldwide, though the incidence of esophageal adenocarcinoma exceeds that of squamous cell carcinoma in the UK, certain other western European countries, and the United States [1]. Smoking and alcohol consumption, known as major risk factors for esophageal squamous cell carcinoma (ESCC), have synergistic effects on carcinogenesis, which are shared with head and neck and also lung cancers [2–4]. Chronic exposure to carcinogens such as nitrosamine related to smoking and the alcohol metabolite aldehyde cause DNA damage and multiple genetic changes [5, 6]. Though no driver gene mutations have yet been detected in ESCC, among solid tumors the somatic mutation rate in ESCC is relatively high [7, 8].

Recent advances in cancer immunology have revealed the importance of signaling between Programmed Death–1 (PD-1), expressed on antigen-experienced T cells, and its ligand PD-L1, expressed on antigen presenting cells and tumor cells (TCs) [9]. Anti-PD-1 or anti-PD-L1 antibodies have clinically benefitted patients with some solid cancers in early clinical trials [10–12]. In a recent phase 3 trial, a favorable response and survival outcomes were obtained with an anti-PD-1 monoclonal antibody, nivolumab, in advanced squamous-cell non-small-cell lung cancer which is genetically similar to ESCC [13, 14]. In addition, a favorable response and durable efficacy of anti-PD-1 monoclonal antibodies for ESCC were also demonstrated in early clinical trials [15, 16]. In the context of biomarker analysis, PD-L1 expression in TCs, that in tumor infiltrating immune cells (TIICs), and a high level of CD8^+^ T cell infiltration have been shown, in several clinical trials testing anti-PD-1 and anti-PD-L1 antibody therapy, to be potential predictive biomarkers of clinical efficacy [17–20].

PD-L1 is expressed on TCs in ESCC and other solid tumors [21, 22]. Recently, high mutation burdens in tumors were reported to be associated with a clinical benefit of PD-1 blockade [23]. Given the relatively high mutation burden in ESCC [7, 8], PD-1/PD-L1 blockade shows promise for treating ESCC. The importance of the immune response to cancer has been studied in terms of infiltration of lymphocytes and macrophages in ESCC [24–26]. To date, no study has explored the associations of PD-L1 expressions in TCs and TIICs and the infiltration of effector cells or immune suppressor cells (regulatory T cells and M2 macrophages).

We performed the present immunohistochemical (IHC) study using surgically resected specimens from a large cohort of treatment-naïve patients with ESCC to identify and quantify PD-L1 expressions in tumors and their associations with anti-tumor immune responses. The survival impacts of various immunological factors were also assessed.

## RESULTS

### PD-L1 expressions in ESCC and TIICs

**Figure 1:**
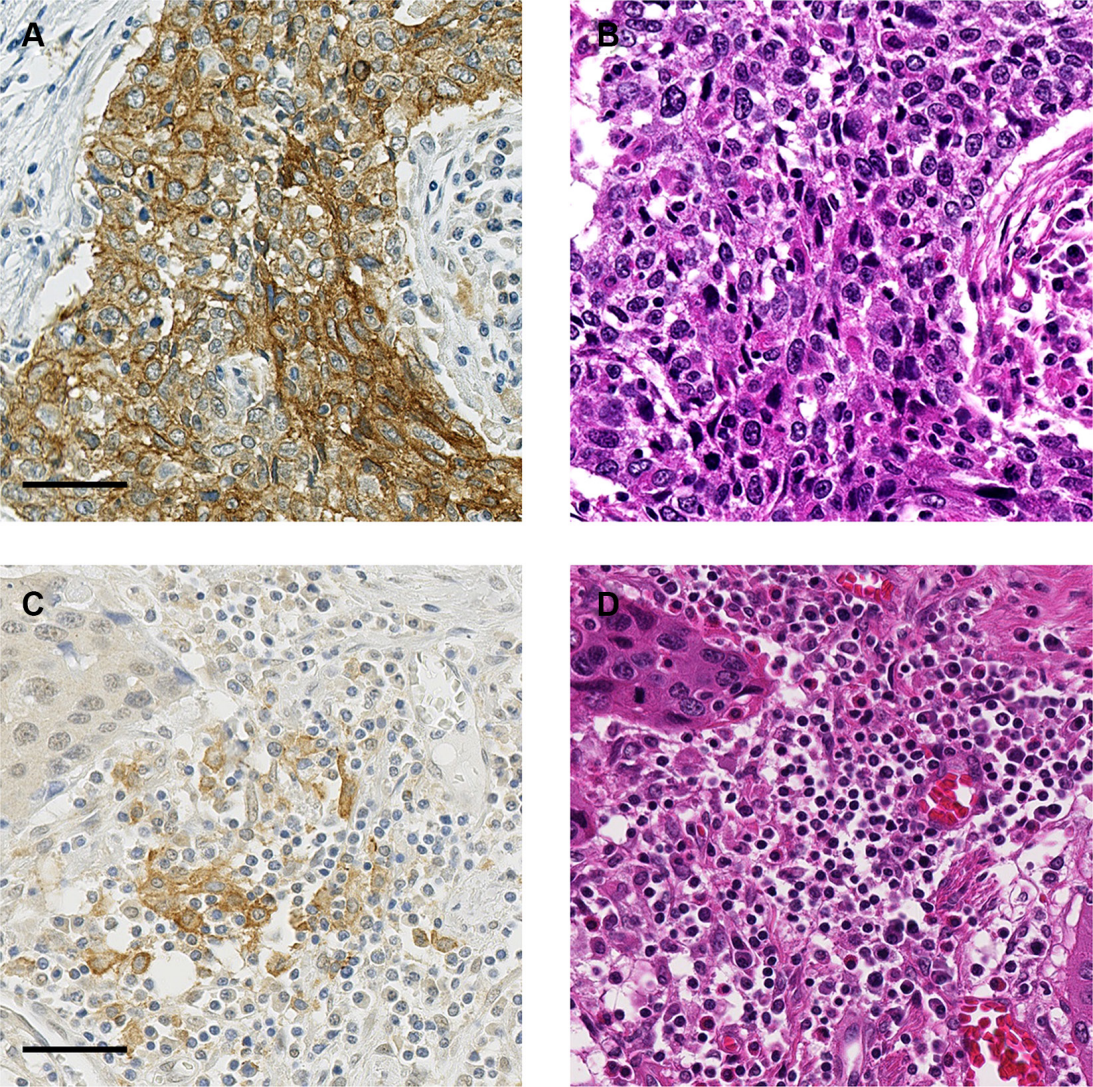
Representative images of PD-L1 expression (**A**) TCs are positive for membranous staining of PD-L1. (**B**) H&E image of the serial section in (A). (**C**) TIICs are positive for PD-L1. (**D**) H&E image of the serial section in (C). Bar: 50 μm.

The clinicopathological characteristics of our patients are listed in Table 1. None of the patients in this study had distant organ metastasis or had received immune therapies, such as immune checkpoint inhibitors and immune cell therapy. Representative cases with PD- L1 expression in TCs and TIICs are shown in Figure 1. PD-L1 expression was positive in TCs from 36 patients (18.4%, 95% confidence interval [CI]: 13.2– 24.5). PD- L1 expression was positive in TIICs from 119 patients (60.7%, 95% CI: 53.5–67.6). Among patients showing PD-L1 positive in TCs, 83.3% demonstrated PD-L1 positive in TIICs, a significantly higher percentage than that in patients whose TCs were PD-L1 negative (*P* < 0.001) (Table 2). In total, 125 patients (63.8%, 95% CI: 56.6–67.6) had TC and/or TIIC positive for PD-L1. The only clinicopathological characteristic significantly associated with PD-L1 expression status, specifically that in TCs, was age (Table 1).

**Table 1:** Clinicopathological characteristics according to PD-L1 expressions in tumor cells and tumor infiltrating immune cells

		Total		Tumor cells					Tumor infiltrating immune cells				
Characteristics		Number	%	Negative	%	Positive	%	*P*	Negative	%	Positive	%	*P*
Age								0.034					0.834
	Median (range)	65 (42–87)		65 (42–83)		68 (53–87)			66 (42–83)		65 (43–87)		
Gender								0.107					0.483
	Male	160	81.6	134	83.8	26	72.2		61	79.2	99	83.2	
	Female	36	18.4	26	16.3	10	27.8		16	20.8	20	16.8	
Smoking status								0.304					0.593
	Non-smoker	42	21.4	32	20.0	10	27.8		15	19.5	27	22.7	
	Smoker	154	78.6	128	80.0	26	72.2		62	80.5	92	77.3	
Alcohol consumption								0.226					0.340
	Non-drinker	26	13.3	19	11.9	7	19.4		8	10.4	18	15.1	
	Drinker	170	86.7	141	88.1	29	80.6		69	89.6	101	84.9	
Location								0.672					0.311
	Upper	24	12.2	21	13.1	3	8.3		9	11.7	15	12.6	
	Middle	78	39.8	62	38.8	16	44.4		26	33.8	52	43.7	
	Lower	94	48.0	77	48.1	17	47.2		42	54.5	52	43.7	
pT								0.831					0.834
	2	32	16.3	25	15.6	7	19.4		13	16.9	19	16.0	
	3	157	80.1	129	80.6	28	77.8		62	80.5	95	79.8	
	4	7	3.6	6	3.8	1	2.8		2	2.6	5	4.2	
pN								0.534					0.858
	0	51	26.0	41	25.6	10	27.8		22	28.6	29	24.4	
	1	59	30.1	49	30.6	10	27.8		24	31.2	35	29.4	
	2	63	32.1	49	30.6	14	38.9		23	29.9	40	33.6	
	3	23	11.7	21	13.1	2	5.6		8	10.4	15	12.6	
pM								0.565					0.228
	0	179	91.3	147	91.9	32	88.9		68	88.3	111	93.3	
	1	17	8.7	13	8.1	4	11.1		9	11.7	8	6.7	
TNM stage								0.916					0.441
	I	7	3.6	6	3.8	1	2.8		4	5.2	3	2.5	
	II	51	26.0	41	25.6	10	27.8		20	26.0	31	26.1	
	III	121	61.7	100	62.5	21	58.3		44	57.1	77	64.7	
	IV	17	8.7	13	8.1	4	11.1		9	11.7	8	6.7	
Grade								0.245					0.794
	W/D	48	24.5	43	26.9	5	13.9		17	22.1	31	26.1	
	M/D	127	64.8	101	63.1	26	72.2		52	67.5	75	63.0	
	P/D	21	10.7	16	10.0	5	13.9		8	10.4	13	10.9	
Lymphatic invasion								0.442					0.668
	Absent	93	47.4	78	48.8	15	41.7		38	49.4	55	46.2	
	Present	103	52.6	82	51.3	21	58.3		39	50.6	64	53.8	
Venous invasion								0.145					0.279
	Absent	24	12.2	17	10.6	7	19.4		7	9.1	17	14.3	
	Present	172	87.8	143	89.4	29	80.6		70	90.9	102	85.7	

Abbreviations: W/D, well differentiated; M/D, moderately differentiated; P/D, poorly differentiated.

**Table 2:** PD–L1 expressions in tumor cells and tumor infiltrating immune cells

	Total	Positive	%	95% CI
TCs	196	36	18.4	13.2–24.5
TIICs	196	119	60.7	53.5–67.6

		TIICs		*P* value (McNemar)
		Negative	Positive	
TCs	Negative	71 (44.4%)	89 (55.6%)	< 0.001
	Positive	6 (16.7%)	30 (83.3%)	

Abbreviations: TCs, tumor cells; TIICs, tumor infiltrating immune cells.

### PD-L1 expression and infiltration of TIICs

**Figure 2:**
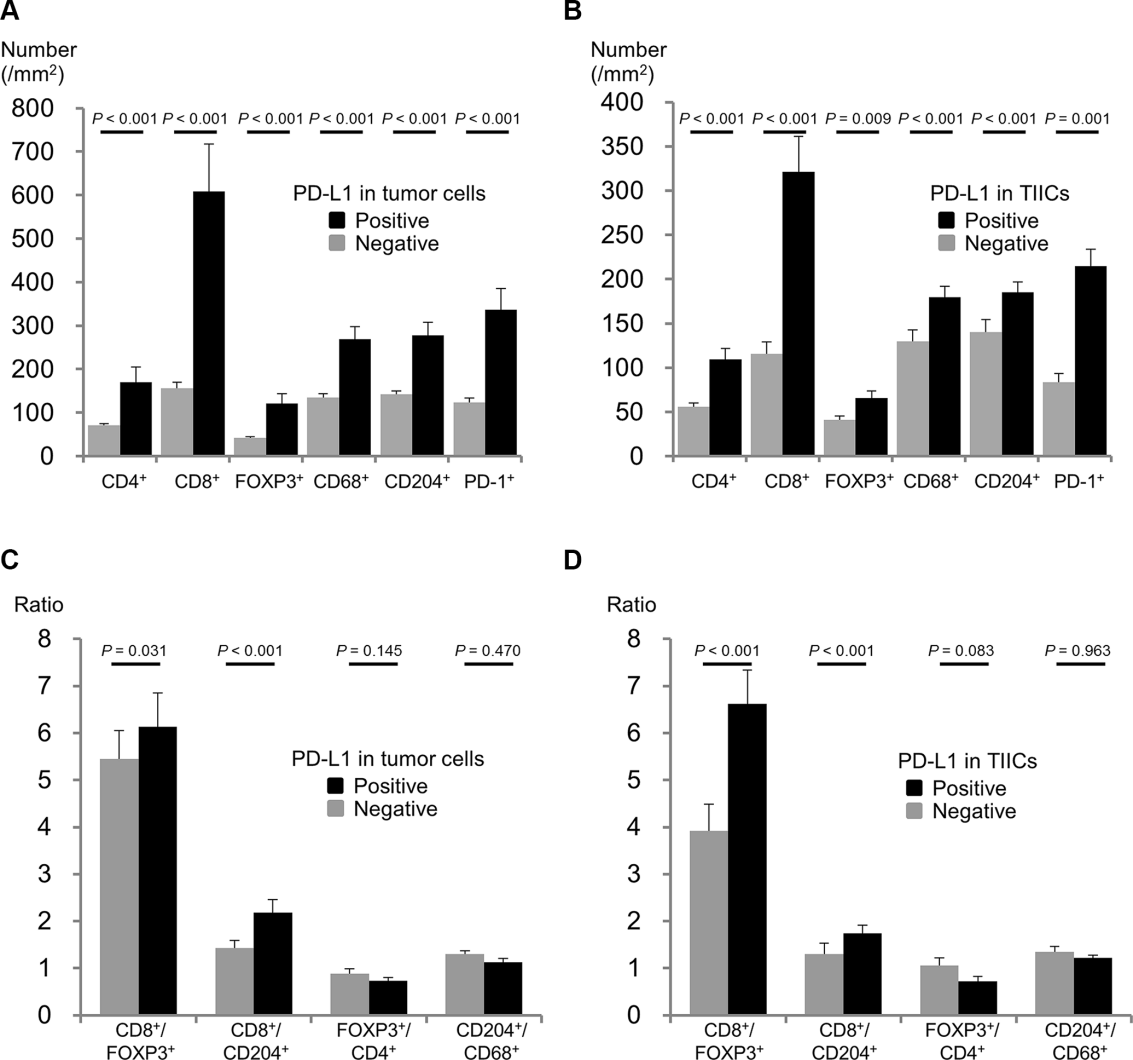
Associations between PD-L1 expression and tumor infiltrating immune cells (**A**) Density of each immune cell type according to PD-L1 expression in TCs. (**B**) Density of each immune cell type according to PD-L1 expression in TIICs. (**C**) Immune cell ratios according to PD-L1 expression in TCs. (**D**) Immune cell ratios according to PD-L1 expression in TIICs.

IHC results of representative cases with each TIIC type are shown in Supplementary Figure S1. Figure 2 demonstrates the relationship between PD-L1 expression status in TCs and TIICs and the number and ratio of each TIIC type. The numbers of all TIIC types evaluated were higher in subgroups with PD-L1 positive in TCs and in TIICs than in subgroups with PD-L1 negative in TCs and in TIICs. This tendency was especially strong for CD8^+^ cells. The CD8^+^/FOXP3^+^ and CD8^+^/CD204^+^ ratios were also higher in subgroups with PD-L1 positive in TCs and in TIICs. In contrast, the FOXP3^+^/CD4^+^ ratio tended to be lower, though not significantly, in subgroups with PD-L1 positive in TCs and in TIICs.

### Survival analyses according to immunological factors

**Figure 3:**
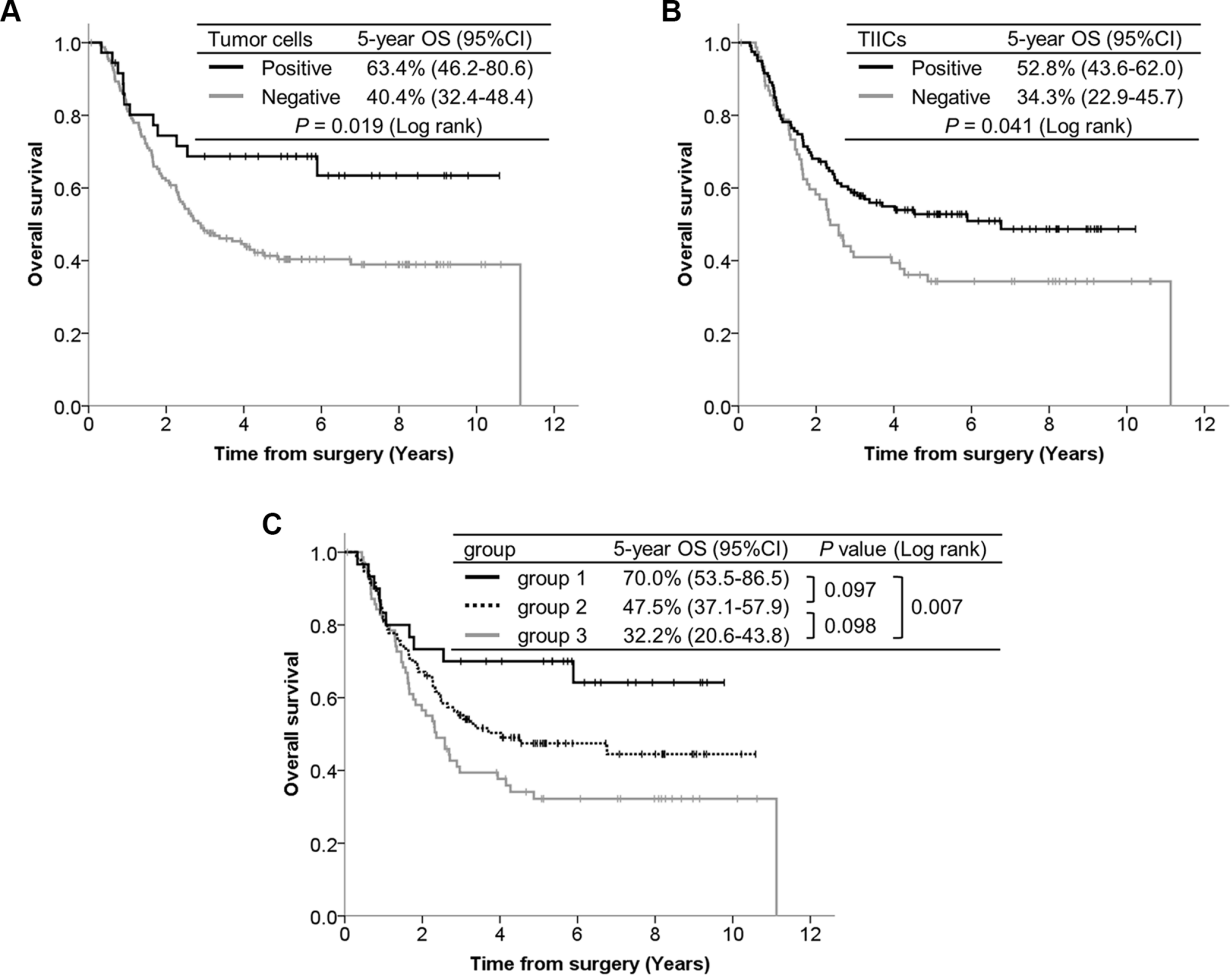
Kaplan-Meier curves according to PD-L1 expression, with 5-year survival rate and the log-rank test for OS (**A**) PD-L1 expression in TCs. (**B**) PD-L1 expression in TIICs. (**C**) Utility of combining PD-L1 expressions in TCs and TIICs; group 1 (*n* = 30): Both TCs and TIICs are PD-L1 positive, group 2 (*n* = 95): either TCs or TIICs are PD-L1 positive, group 3 (*n* = 71): both TCs and TIICs are PD-L1 negative.

The median follow-up time of the censored cases was 5.5 years (range, 0.1–10.6) from the date of surgery. The overall survival (OS) curves according to PD-L1 expression are presented in Figure 3. Patients with PD-L1 positive in TCs demonstrated significantly better OS than those with PD-L1 negative in TCs (*P* = 0.019). Patients with PD-L1 positive in TIICs had significantly better OS than those with PD-L1 negative in TIICs (*P* = 0.041). We divided patients into three groups according to the PD- L1 expression status of their TCs and TIICs: both PD-L1 positive (group 1), either one PD-L1 positive (group 2), and both PD-L1 negative (group 3). The separate OS curves for these 3 groups revealed a statistically significant difference only between groups 1 and 3 (*P* = 0.097 between groups 1 and 2, *P* = 0.098 between groups 2 and 3, *P* = 0.007 between groups 1 and 3).

**Figure 4:**
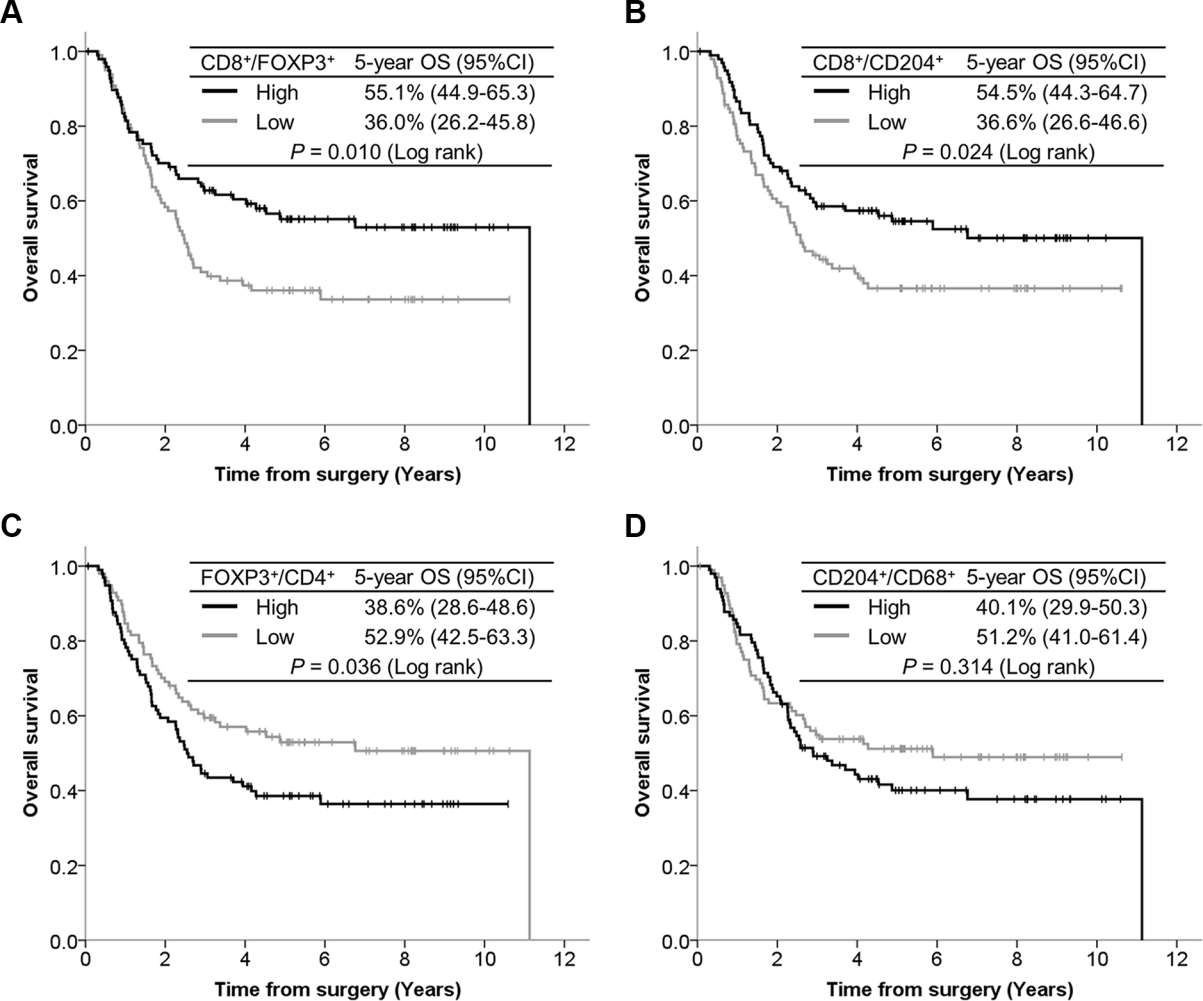
Kaplan-Meier curves according to TIIC ratios, with 5-year survival rate and the log-rank test for OS (**A**) CD8^+^/FOXP3^+^ ratio. (**B**) CD8^+^/CD204^+^ ratio. (**C**) FOXP3^+^/CD4^+^ ratio. (**D**) CD204^+^/CD68^+^ ratio.

The OS curves according to TIIC ratios are presented in Figure 4. Patients with a high CD8^+^/FOXP3^+^ ratio had significantly better OS than those with a low CD8^+^/FOXP3^+^ ratio (*P* = 0.010). Patients with a high CD8^+^/CD204^+^ ratio had significantly better OS than those with a low CD8^+^/CD204^+^ ratio (*P* = 0.024). In contrast, patients with a high FOXP3^+^/CD4^+^ ratio showed significantly poorer OS than those with a low FOXP3^+^/CD4^+^ ratio (*P* = 0.036). Although the OS curves were drawn separately according to CD204^+^/CD68^+^ ratios, the difference did not reach statistical significance (*P* = 0.314). Among each TIIC type assessed, prognostic significance was demonstrated in CD8^+^ cells (*P* = 0.040) and PD-1^+^ cells (*P* = 0.032) (Supplementary Figure S2).

### Multivariate analyses for survival outcomes

The results of univariate and multivariate survival analyses are presented in Table 3. Both TCs (HR 0.461, 95% CI: 0.246–0.864, *P* = 0.016) and TIICs (HR 0.590, 95% CI: 0.394–0.884, *P* = 0.010) being PD-L1 positive was significantly associated with longer OS in multivariate analysis even after adjusting for potentially confounding clinicopathological factors. A high CD8^+^/FOXP3^+^ ratio (HR 0.617, 95% CI: 0.413–0.923, *P* = 0.019) and a high CD8^+^/CD204^+^ ratio (HR 0.650, 95% CI: 0.439–0.962, *P* = 0.031) were also significantly associated with longer OS, although none of the TIIC types individually showed a significant association with OS except for PD-1^+^ cells (HR 0.579, 95% CI: 0.387–0.865, *P* = 0.008). We explored OS based on TIICs in the stroma by focusing on factors significantly impacting OS in the tumor nest assessment (CD8^+^/FOXP3^+^, CD8^+^/CD204^+^, and PD-1^+^). None significantly impacted OS in multivariate analyses (Supplementary Figure S3).

**Table 3:** Univariate and multivariate Cox regression analyses for overall survival

Univariate analysis			Overall survival		
Clinicopathological factors		ref	HR	95% CI	*P*
Age	> 65	≤ 65	0.866	0.588–1.274	0.465
Gender	Female	Male	0.771	0.452–1.314	0.339
Smoking status	Smoker	Non-smoker	1.355	0.815–2.252	0.242
Alcohol consumption	Drinker	Non-drinker	0.969	0.541–1.733	0.914
Location	lower	Upper/Middle	0.893	0.606–1.315	0.566
pT factor	4	2–3	1.600	0.587–4.361	0.358
LN metastasis	Present	Absent	3.274	1.827–5.867	< 0.001
Histological grade	P/D	W/D, M/D	1.857	1.073–3.213	0.027
Lymphatic invasion	Present	Absent	2.180	1.454–3.269	< 0.001
Venous invasion	Present	Absent	1.628	0.848–3.125	0.143

Immunological factors		ref	HR	95% CI	*P*
PD-L1 (TCs)	Positive	Negative	0.493	0.270–0.901	0.022
PD-L1 (TIICs)	Positive	Negative	0.670	0.455–0.986	0.042
CD4	High	Low	0.786	0.535–1.154	0.219
CD8	High	Low	0.668	0.454–0.984	0.041
FOXP3	High	Low	1.279	0.868–1.885	0.213
PD-1	High	Low	0.656	0.445–0.967	0.033
CD68	High	Low	0.877	0.597–1.289	0.505
CD204	High	Low	1.239	0.842–1.824	0.276
CD8^+^/FOXP3^+^	High	Low	0.601	0.406–0.890	0.011
CD8^+^/CD204^+^	High	Low	0.641	0.435–0.946	0.025
FOXP3^+^/CD4^+^	High	Low	1.509	1.023–2.225	0.038
CD204^+^/CD68^+^	High	Low	1.219	0.828–1.795	0.315

Multivariate analysis^#^			Overall survival		
Immunological factors		ref	HR	95% CI	*P*
PD-L1 (TCs)	Positive	Negative	0.461	0.246–0.864	0.016
PD-L1 (TIICs)	Positive	Negative	0.590	0.394–0.884	0.010
CD4	High	Low	0.787	0.527–1.175	0.599
CD8	High	Low	0.693	0.456–1.052	0.085
FOXP3	High	Low	1.267	0.837–1.918	0.263
PD-1	High	Low	0.579	0.387–0.865	0.008
CD68	High	Low	1.009	0.668–1.526	0.964
CD204	High	Low	1.299	0.866–1.964	0.206
CD8^+^/FOXP3^+^	High	Low	0.617	0.413–0.923	0.019
CD8^+^/CD204^+^	High	Low	0.650	0.439–0.962	0.031
FOXP3^+^/CD4^+^	High	Low	1.381	0.922–2.067	0.117
CD204^+^/CD68^+^	High	Low	1.106	0.742–1.648	0.621

Abbreviations: HR, hazard ratio; CI, confidence interval; LN, lymph node; W/D, well differentiated; M/D, moderately differentiated; P/D, poorly differentiated;TCs, tumor cells; TIICs, tumor infiltrating immune cells.

# adjusted for age, gender, smoking habit, alcohol consumption, pT factor, LN metastasis, histological grade, lymphatic invasion, and venous invasion.

## DISCUSSION

We clarified PD-1 and PD-L1 expressions, the associations between PD-L1 expression and various immune cells, and the prognostic relevance of these factors employing IHC with tissue microarrays for 196 ESCC cases who had received neither preoperative neoadjuvant therapy nor post-recurrence immunotherapy.

Reports of PD-L1 expression in ESCC are limited. PD-L1 positive rates in TCs were reported to be 41.9% by Ohigashi et al. and 84.5% by Chen et al. [21, 22], though PD-L1 expressions on the plasma membrane and in the cytoplasm were defined as positive in their studies. Mechanistically, PD-L1 is a type I transmembrane molecule expressed on TCs and binds to its receptor, PD-1, which is expressed on the plasma membranes of activated T cells [27]. Furthermore, PD-L1 expression on the plasma membrane has been evaluated as a biomarker candidate in recent clinical trials of anti-PD-1 or anti-PD-L1 antibody therapy [17–19]. Accordingly, we evaluated PD-L1 expression on the plasma membranes of TCs employing a cut-off value of 1% based on the sensitivity test assessing hazard ratios for OS, and demonstrated the positive rate to be 18.4% in our patients. The positive rate of 63.8% for PD-L1 expression in TCs and/or TIICs demonstrated herein was compatible with the results of a phase 1 trial for esophageal cancer [15].

Marked infiltration of CD8^+^ cells into a PD-L1 positive tumor has been reported for several cancers including ESCC [21, 28, 29]. In this study, abundant infiltrations of CD8^+^ and PD-1^+^ cells were associated with PD-L1 expression in TCs and TIICs, observations in line with the theory that PD-L1 expression is induced by several pro-inflammatory factors, such as IFNγ and TNFα, produced by activated T cells [30, 31], and that the PD-1/PD-L1 pathway plays a role in suppressing activated T cells in the periphery. In addition, marked infiltrations of immune suppressor cells such as regulatory T cells (FOXP3^+^) and M2 macrophages (CD204^+^) were also associated with PD-L1 expression in both TCs and TIICs. These results indicate that PD-L1 expression in TCs and/or TIICs reflects a highly activated immune response to tumors and also the adaptive immune resistance which develops as a consequence. In contrast, significant associations of positivity for PD-L1 expression with the CD8^+^/FOXP3^+^ and CD8^+^/CD204^+^ ratios, as well as a trend for an inverse association of PD-L1 expression with the FOXP3^+^/CD4^+^ ratio, were observed, suggesting PD- L1 expression to be associated with the balance between infiltrating effector cells and immune suppressor cells.

The prognostic significance of PD-L1 expression in TCs remains controversial. Previous studies of ESCC demonstrated an association between PD-L1 expression and poor outcomes [21, 22]. However, we found PD-L1 expression to be a factor predicting favorable OS. Similar discordant results have also been reported for other cancers, such as melanoma and lung cancer [32–36]. We speculate that this discrepancy regarding prognostic relevance between the present and previous studies may be due not only to the definitions of positive staining applied, but also differences in the antibodies used and heterogeneous baseline characteristics. We also revealed PD-L1 expression in TIICs and high infiltration of PD-1^+^ TIICs to predict favorable OS, which is compatible with recent reports on several cancer types [29, 37–40]. PD-1 and PD-L1 are inhibitory immune checkpoint molecules. However, considering that PD-1 is expressed mainly on activated T cells and PD-L1 expression is induced by activated T cells, and also the association between PD- L1 expression and TIIC abundance including PD-1^+^ cells, PD-1 and PD-L1 expressions should be regarded as reflecting an immunoreactive state, which contributes to better OS [9]. PD-L1 positive tumors, which generally have abundant TIICs including PD-1^+^ immune cells, may define a subset of ESCC patients who are potential candidates for anti-PD-1 or anti-PD-L1 antibody therapy. However, we cannot draw a conclusion based on our present results as this study did not include patients receiving these agents. Ongoing clinical trials of these agents are anticipated to clarify the optimal predictive biomarkers.

The number of tumor infiltrating FOXP3^+^ regulatory T cells, especially as reflected by a decreased CD8^+^/FOXP3^+^ ratio, is reportedly associated with a poor prognosis for several cancer types [41–44]. In addition to confirming these prior reports, our results clarified the prognostic significance of the CD8^+^/CD204^+^ ratio. CD204^+^ macrophages are another set of immune suppressor cells (M2 phenotype) [45], and marked CD204^+^ macrophage infiltration is reportedly associated with a malignant phenotype or poor survival for several cancers including ESCC [26, 46–48]. Given the functions of M2 macrophages, such as producing immune suppressive cytokines and downregulating effector T cell activity [49, 50], not only the number of CD204^+^ cells but also the balance between CD8^+^ and CD204^+^ cells must be considered. Our study is the first, to our knowledge, to demonstrate the positive survival impact of an increased CD8^+^/CD204^+^ ratio. These results suggest that regulatory T cells and M2 macrophages play a critical role in ESCC progression, making these cell types potentially novel therapeutic targets for agents which could be used in addition to immune checkpoint inhibitors targeting PD-1/PD-L1 signaling.

Herein, TIICs in the stroma were not associated with OS. The clinical relevance of TIIC localization, whether in the tumor nest or the stroma, remains controversial. Although several reports have demonstrated the importance of the stroma [51–53], our results indicate that tumor nest TIICs have more clinical relevance in ESCC. Tumor microenvironments may differ among cancer types. The present results are understandable, considering that tumor nest TIICs are in direct contact with TCs. A report on melanoma showed lymphocytes within the tumor nests to be remarkably increased after immune checkpoint inhibitor treatment in patients who demonstrated a response, suggesting the importance of tumor nest TIICs [54].

We examined for the first time the impacts of various immunological factors in both TCs and TIICs from a large cohort of patients with pure ESCC. Although we enrolled 196 patients, a considerable number, this study was performed retrospectively, in a single institution. A prospective study is needed to validate the present results.

In summary, we employed comprehensive IHC analyses in patients with ESCC. We demonstrated PD- L1 expression in a significant proportion of patients with ESCC, its association with marked infiltration of TIICs, the prognostic significance of PD-1 and PD-L1 expressions and the impact on clinical outcomes of the balance between infiltrating effector cells and immune suppressor cells. Given the complex network constituting anti-tumor immunity, such comprehensive analyses should be applied when assessing immune status in the tumor microenvironment.

## MATERIALS AND METHODS

### Patients and specimens

Among the 372 patients with no prior therapy who underwent surgical resection of esophageal cancer between 2000 and 2011 at the National Cancer Center Hospital East, Kashiwa, Japan, 196 were consecutively enrolled in this study based on the following selection criteria: i) histologically confirmed squamous cell carcinoma, ii) pathological T factor of at least T2 according to the TNM classification [55], iii) complete resection performed, iv) no in-hospital death after surgery, and v) sufficient formalin-fixed paraffin-embedded surgically resected tissue sample amounts available. Clinical and pathological information was collected from medical records including the pathology report for each subject. The study protocol was approved by the institutional review board of the National Cancer Center in October 2014 (2014–124). The study was carried out according to the Epidemiological Study Guideline of the Ministry of Health, Labour and Welfare of Japan. We disclosed the study design on the National Cancer Center website and gave the relatives of deceased patients the opportunity to decline participation.

After reviewing hematoxylin and eosin (H&E) slides of the archived primary tumors, a representative block was selected in each case. A 2.0-mm in diameter tumor core was obtained from the center of the selected block, using a manual tissue arrayer (Azumaya Ika Kikai, Tokyo, Japan). These cores were assembled in a tissue microarray (TMA) format, and paraffin-embedded TMA blocks were then cut into 4-mm sections and placed on silicon-coated slides for IHC staining.

### Immunohistochemistry

The primary antibodies used for IHC and the IHC assay are described in Supplementary Table S1. For CD4, CD8 and CD68, IHC was performed employing ready-to-use antibodies and the fully automated Ventana Benchmark ULTRA platform (Ventana, Tucson, AZ, USA) according to the manufacturer's instructions. For CD204, IHC was performed using the Ventana Benchmark ULTRA platform semi-automatically with manual application of the primary antibody. For FOXP3, PD-1 and PD-L1, IHC was performed manually.

### Evaluation of PD-L1 expression and tumor infiltrating immune cells

After IHC, the slides were scanned and the microscopic images were imported as digital photo files using the NanoZoomer Digital Pathology (NDP) system (Hamamatsu Photonics, Hamamatsu, Japan). PD-L1 expression was identified by two independent observers (KH and SF) blinded to all of the clinical data. To assess PD-L1 in TCs, the proportion of TCs with membrane staining was scored as < 1%, 1–4%, 5–9%, 10%, and then at 10% intervals up to ≥ 50%. When the difference between the assessments of the two pathologists was one level or greater, the slide was reviewed jointly and a single consensus score was established. Finally, PD-L1 positive expression in TCs was defined as the presence of ≥ 1% of TCs with membrane staining based on the hazard ratio for OS (Supplementary Figure S1). PD-L1 expression in TIICs was determined qualitatively to be either positive (any expression of PD-L1 in TIICs in the core) or negative (no staining).

To quantitatively evaluate each TIIC type, the entire tumor core was reviewed using NDP view at a magnification of × 200 and 4 independent areas with a size of 0.0625 mm^2^, containing the greatest abundance of TIICs in the tumor nest, were selected. After counting the TIICs in each selected area using NDP view at a magnification of × 400, numbers of the respective TIICs per square millimeter calculated from the total number in the 4 selected areas were presented. We calculated ratios of infiltrating effector cells to immune suppressor cells, such as CD8^+^/FOXP3^+^ (CD8^+^ cell count divided by FOXP3^+^ cell count) and CD8^+^/CD204^+^, and ratios of the infiltrating immune suppressor cells to the corresponding whole cell populations, such as FOXP3^+^/CD4^+^ and CD204^+^/CD68^+^, were also determined.

### Statistical analysis

The numbers of each TIIC type were compared according to PD-L1 expression status (positive/negative), in TCs and TIICs, using the Mann-Whitney *U* test. The Chi-square test was applied to assess the relationships between clinicopathological factors and PD-L1 expressions in TCs and TIICs, and the McNemar test was used to compare the proportions positive for PD-L1 expression between TCs and TIICs. OS was defined as the period from the date of surgery until the date of death from any cause. Patients were censored at the time of their last follow-up, if they were alive. OS rates were estimated using the Kaplan-Meier method, and were compared using the log-rank test. Immunological factors other than PD-L1 expression status were dichotomized according to their median values (≥ median/< median).

Univariate and multivariate Cox proportional hazards models were used to examine the associations of clinicopathological factors with OS. The impacts of immunological factors (PD-L1 expressions in TCs and TIICs, number of each type of TIIC, and TIIC ratios [CD8^+^/FOXP3^+^, CD8^+^/CD204^+^, FOXP3^+^/CD4^+^ and CD204^+^/CD68^+^]) on OS were first examined with univariate Cox regression models. Next, the impact of each immunological factor on OS was assessed by adding each factor to the multivariate model which contained the possibly confounding clinicopathological factors such as age, gender, smoking status, alcohol consumption, location, pT factor, lymph node metastasis, histological grade, lymphatic invasion, and venous invasion. All statistical analyses were performed using IBM SPSS statistics 20 (IBM Japan Ltd., Tokyo, Japan). All *P* values were two-sided, with a significance level of 0.05.

## SUPPLEMENTARY MATERIALS


