# Supplementary material for: Comprehensive immunohistochemical analysis of tumor microenvironment immune status in esophageal squamous cell carcinoma
Source: Oncotarget. 2016 Jun 15;7(30):47252–64. doi: 10.18632/oncotarget.10055 (PMC5216939; doi:10.18632/oncotarget.10055)
Supplement: Supplementary file 1 [file oncotarget-07-47252-s001.pdf]

Comprehensive immunohistochemical analysis of tumor microenvironment immune status in esophageal squamous cell carcinoma

Supplementary Materials

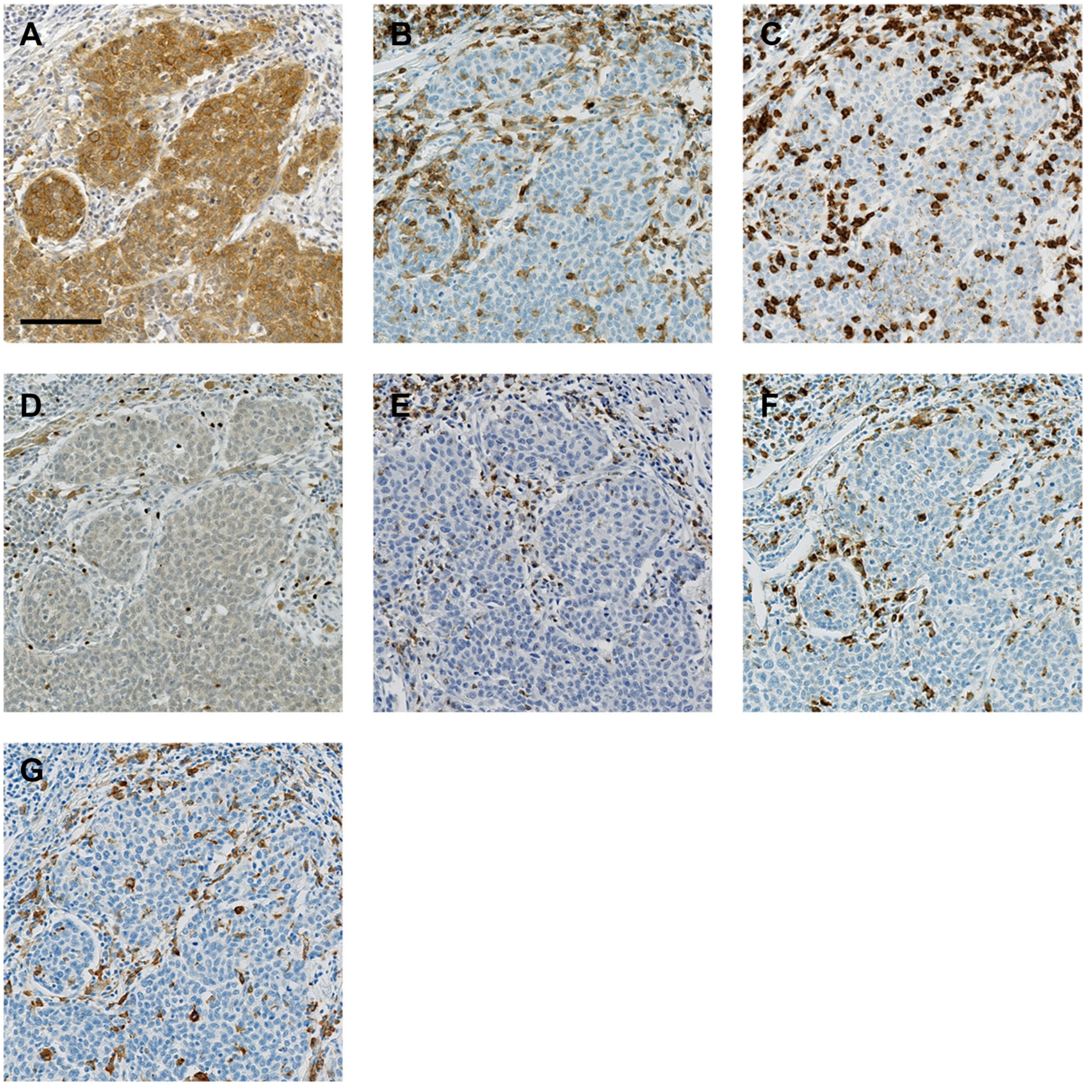

Supplementary Figure S1: Representative IHC images of PD-L1 expression and each tumor infiltrating immune cell type in serial sections. (**A**) PD-L1 positive tumor. (**B**) $CD4^+$ cells. (**C**) $CD8^+$ cells. (**D**) $FOCP3^+$ cells. (**E**) PD-1^+ cells. (**F**) $CD68^+$ macrophages. (**G**) $CD204^+$ macrophages. Bar: 100 µm.

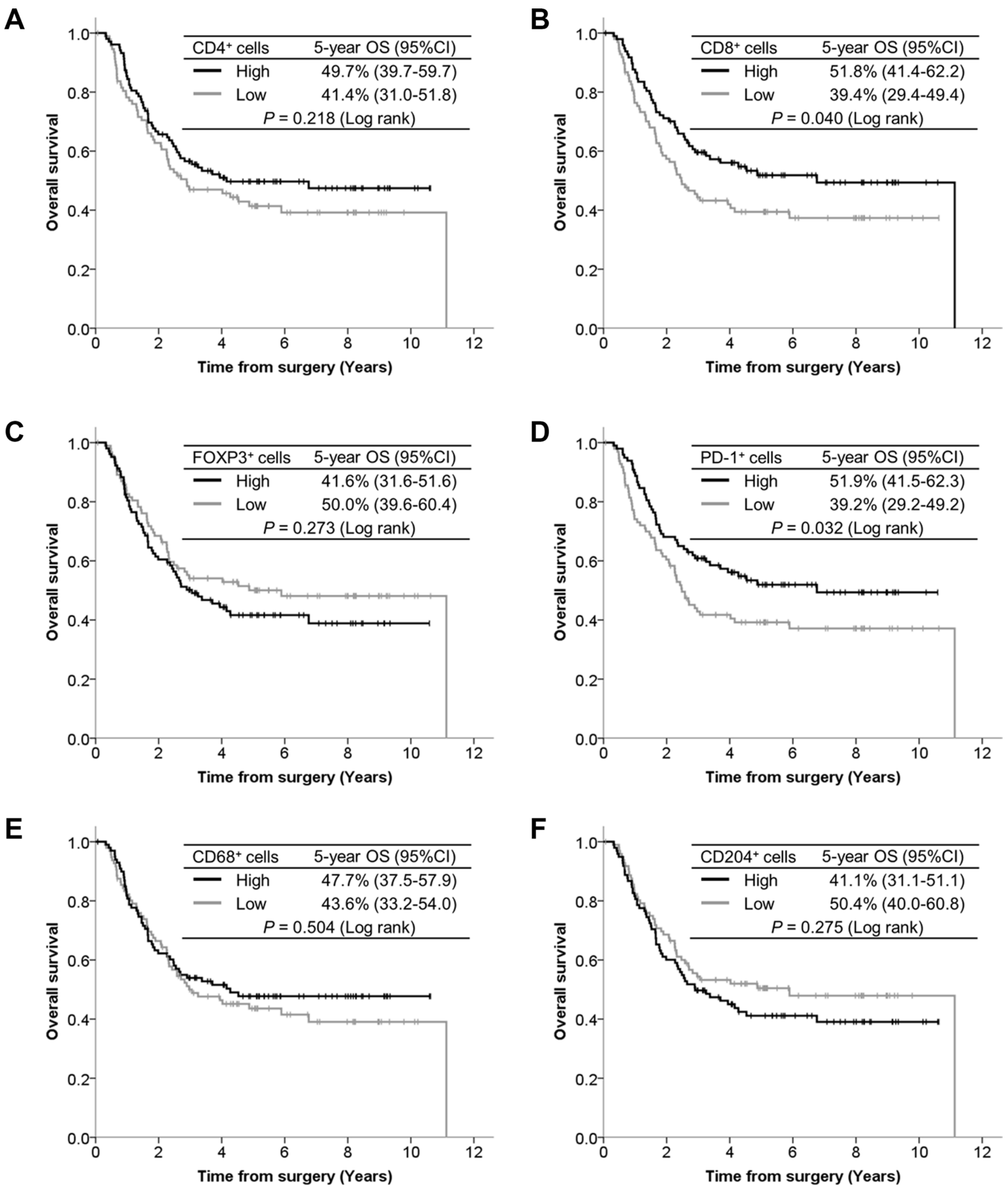

Supplementary Figure S2: Kaplan-Meier curves according to each of the TIIC types with 5-year survival rate and the log-rang test for OS. (**A**) $CD4^+$ cells. (**B**) $CD8^+$ cells. (**C**) $FOCP3^+$ cells. (**D**) PD-1^+ cells. (**E**) $CD68^+$ cells. (**F**) $CD204^+$ cells.

A

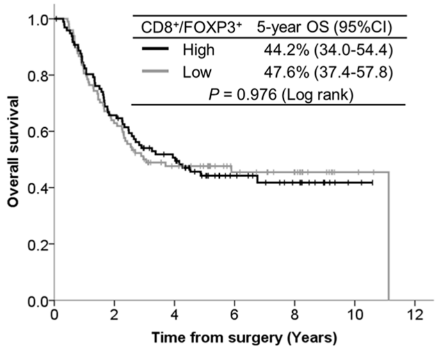

B

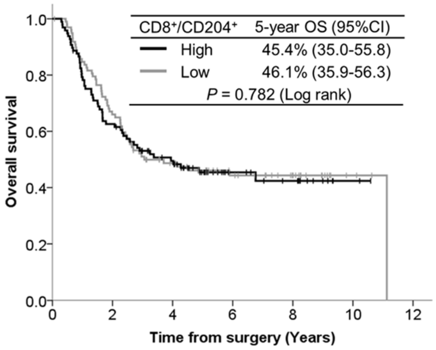

C

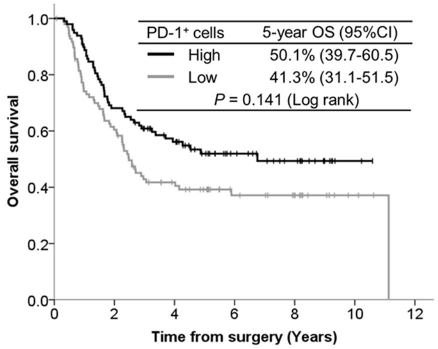

D

Multivariate analysis#			Overall survival		
Immunological factors		ref	HR	95% CI	*P*
$CD8^+/FOXP3^+$	High	Low	0.874	0.584-1.307	0.511
$CD8^+/CD204^+$	High	Low	1.161	0.780-1.729	0.462
PD-1	High	Low	0.751	0.497-1.135	0.174

Abbreviations: HR, hazard ratio; CI, confidence interval; LN, lymph node
#adjusted for age, gender, smoking habit, alcohol consumption, pT factor, LN metastasis, histological grade, lymphatic invasion, and venous invasion

Supplementary Figure S3: Survial analyses according to TIICs in the tumor stroma. Kaplan-Meier curves of OS for (**A**) $CD8^+/FOXP3^+$ ratio, (**B**) $CD^+/CD204^+$ ratio, and (**C**) $PD\text{-}1^+$ cells. (**D**) Multivariate Cox regression analyses for OS.

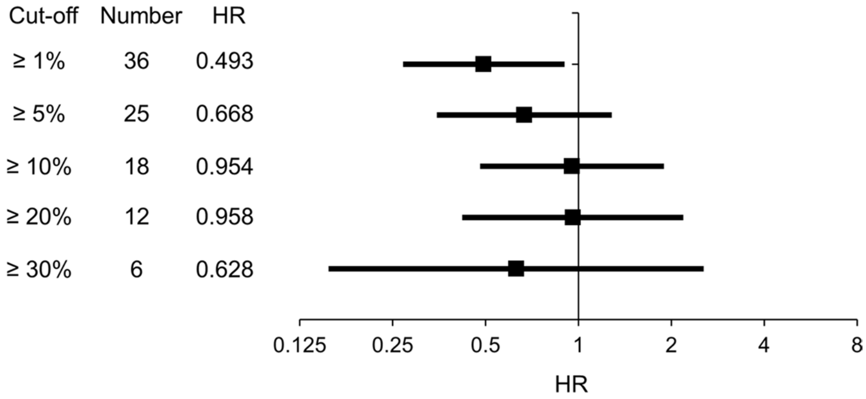

Supplementary Figure S4: Hazard ratio for OS according to the cut-off value for PD-L1 expression in tumor cells. A sensitivity test was performed using the hazard ratio for OS with the cut-off values for PD-L1 expression in cancer cells according to the proportions of tumor cells with PD-L1 membrane stained scored 1%, 5%, 10%, 20%, and 30%. The minimum hazard ratio was obtained when the Ccut-off value of 1% was adopted (0.493, 95% CI: 0.270–0.901, $P = 0.022$).

Supplementary Table S1: Antibodies and immunohistochemical assays

Marker	Source	Type	Clone	Procedure	Dilution	Antigen retrieval	Visualization	Manufacturer
PD-L1	Rabbit	Monoclonal	E1L3N	Manual	1:400	M/W (95°C, 10 min.), Citrate buffer (pH 6.0)	Standard DAB procedure	Cell Signaling Technology (Cambridge, UK)
CD4	Rabbit	Monoclonal	SP35	Autostainer	Ready-to-use	Heat (95°C, 64 min.), CC1: EDTA buffer (pH 8.5)	iVIEW DAB Detection Kit	Ventana (Tucson, AZ, USA)
CD8	Rabbit	Monoclonal	SP57	Autostainer	Ready-to-use	Heat (95°C, 64 min.), CC1: EDTA buffer (pH 8.5)	ultra View Universal DAB Detection Kit	Ventana
FOXP3	Mouse	Monoclonal	236A/E7	Manual	1:100	A/C (121°C, 10 min.), Citrate buffer (pH 6.0)	Standard DAB procedure	Abcam (Cambridge, UK)
PD-1	Mouse	Monoclonal	EH33	Manual	1:200	M/W (95°C, 20 min.), EDTA buffer (pH 9.0)	Standard DAB procedure	Cell Signaling Technology
CD68	Mouse	Monoclonal	KP-1	Autostainer	Ready-to-use	Heat (95°C, 64 min.), CC1: EDTA buffer (pH 8.5)	ultra View Universal DAB Detection Kit	Ventana
CD204	Mouse	Monoclonal	SRA-E5	Autostainer	1:400	Heat (95°C, 64 min.), CC1: EDTA buffer (pH 8.5)	iVIEW DAB Detection Kit	Transgenic (Kumamoto, Japan)

Abbreviations: M/W, microwave; EDTA, ethylenediaminetetraacetic acid; DAB, diaminobenzidine.
For FOXP3, and PD-L1, slides were dewaxed and rehydrated in distilled water, and endogenous peroxidase activity was then blocked by immersion in 3% hydrogen peroxide in methanol for 10 minutes. After antigen retrieval, the slides were incubated overnight at 4°C with each primary antibody. The slides were then further incubated with anti-mouse secondary antibody (EnVision+ System-HRP Labelled Polymer Anti-mouse, Dako, Tokyo, Japan) for FOXP3 and PD-1 or anti-rabbit secondary antibody (EnVision+ System-HRP Labelled Polymer Anti-rabbit, Dako) for PD-L1, and staining was detected using a standard diaminobenzidine procedure. Finally, the sections were counterstained with hematoxylin.
